# Emerging Autophagy Functions Shape the Tumor Microenvironment and Play a Role in Cancer Progression - Implications for Cancer Therapy

**DOI:** 10.3389/fonc.2020.606436

**Published:** 2020-11-25

**Authors:** Silvina Odete Bustos, Fernanda Antunes, Maria Cristina Rangel, Roger Chammas

**Affiliations:** Instituto do Cancer do Estado de São Paulo, Faculdade de Medicina de São Paulo, Brazil

**Keywords:** tumor microenvironment, secretion, immune system, epithelial-mesenchymal transition, cancer, new autophagy functions

## Abstract

The tumor microenvironment (TME) is a complex environment where cancer cells reside and interact with different types of cells, secreted factors, and the extracellular matrix. Additionally, TME is shaped by several processes, such as autophagy. Autophagy has emerged as a conserved intracellular degradation pathway for clearance of damaged organelles or aberrant proteins. With its central role, autophagy maintains the cellular homeostasis and orchestrates stress responses, playing opposite roles in tumorigenesis. During tumor development, autophagy also mediates autophagy-independent functions associated with several hallmarks of cancer, and therefore exerting several effects on tumor suppression and/or tumor promotion mechanisms. Beyond the concept of degradation, new different forms of autophagy have been described as modulators of cancer progression, such as secretory autophagy enabling intercellular communication in the TME by cargo release. In this context, the synthesis of senescence-associated secretory proteins by autophagy lead to a senescent phenotype. Besides disturbing tumor treatment responses, autophagy also participates in innate and adaptive immune signaling. Furthermore, recent studies have indicated intricate crosstalk between autophagy and the epithelial-mesenchymal transition (EMT), by which cancer cells obtain an invasive phenotype and metastatic potential. Thus, autophagy in the cancer context is far broader and complex than just a cell energy sensing mechanism. In this scenario, we will discuss the key roles of autophagy in the TME and surrounding cells, contributing to cancer development and progression/EMT. Finally, the potential intervention in autophagy processes as a strategy for cancer therapy will be addressed.

## Introduction

The autophagy process has been explored for almost 60 years, from morphological studies since early 70’s to molecular studies initiated in the 1990s (1–3). During this period, several studies were conducted to understand the genetic mechanisms of autophagy, leading to the discovery of autophagy-related genes (ATG) in yeast (4, 5). Subsequently, ATG homologs were identified in various organisms, and new ATG genes were described in mammals (6). Building upon these findings, efforts to delve into the molecular mechanisms involved in the degradation of intracellular constituents have grown rapidly. However, several issues remain unsolved regarding the molecular regulation of autophagy, its integration and control at the tissue and systemic levels and its role in cancer pathophysiology. Three main types of autophagy have been described, depending on the morphology and mechanisms: microautophagy, chaperone-mediated autophagy and the best characterized macroautophagy (hereafter referred as autophagy).

**Figure 1:**
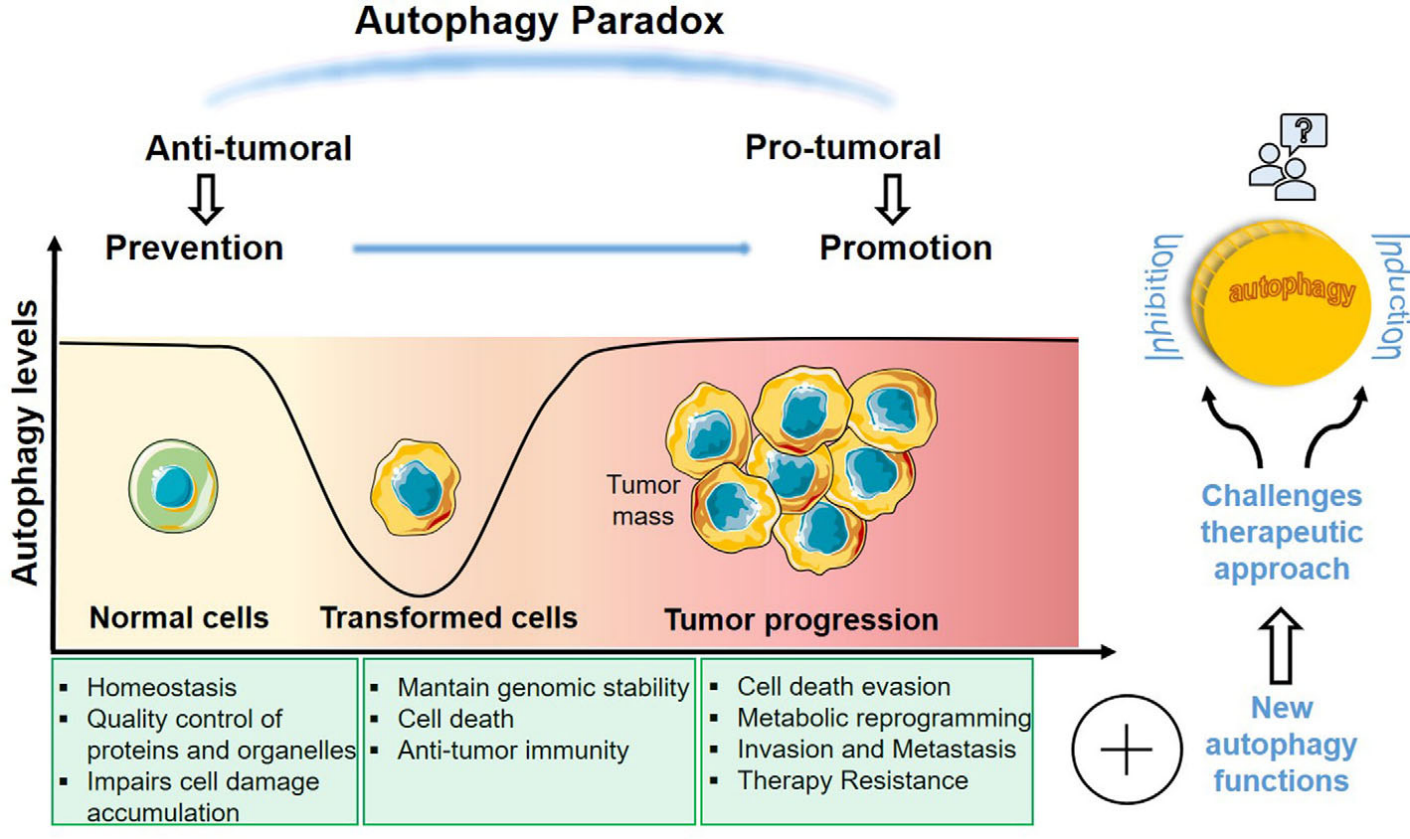
Dual role of autophagy in cancer. Autophagy is implicated in several stages of tumorigenesis executing different processes. The extensive and opposite functions in cancer makes autophagy an important target to develop new therapies. A deeper knowledge about this complex feature of autophagy in cancer research is essential to find more accurate therapeutic approaches.

Autophagy is an evolutionarily conserved process responsible for removing intracellular molecular aggregates of misfolded proteins and damaged organelles, through the sequestration of these substrates in a double-membrane vesicle, which fuses with lysosomes, where degradation of the macromolecular machines or complexes takes place. Multiple proteins are involved in the sequential stages of autophagy consisting in initiation, elongation of isolated membranes decorated by microtubule-associated protein 1A/1B-light chain 3 (LC3) to form an autophagosome, and its fusion with the lysosome to cargo degradation. Autophagy is stimulated in physiological and pathological conditions regulating cell metabolism and homeostasis (7). In cancer cells, stressors, such as hypoxia or nutrient deprivation, induce autophagy to support the high energy demand of cells with dysregulated proliferation. Many tumor suppressor and oncogene products are elements of autophagy pathways, pointing to the relationship between autophagy and tumorigenesis. Moreover, it is well known that many cancer cells have high basal levels of autophagy. Although autophagy contributes to cancer promotion in advanced stages, it is also capable to inhibit tumor initiation in early stages (8, 9). The molecular circuitry controlling autophagy is therefore complex, as it can either induce cell-death or promote cell survival (**Figure 1**) (10, 11). Understanding the mechanisms for the protective role of autophagy in cancer is essential for the identification of novel targets to the control of resistance of tumors to treatment.

The process of recycling cellular components performed by autophagy has been well characterized. Beyond self-eating and recycling damaged organelles, new roles for autophagy and the ATG genes have been ascribed (12, 13). Indeed, autophagy interferes in a wide range of cellular processes. Interestingly, components of autophagy can influence dynamic cellular processes and lead to tumor microenvironment (TME) reprogramming. Here, we discuss the novel roles of the autophagy machinery in tumor secretion, immune response, migration, and invasion capacity of tumor cells undergoing the epithelial to mesenchymal transition (EMT) (14, 15). These processes may occur simultaneously or not, affecting not only tumor cells, but also tumor microenvironmental components. These processes may also be interconnected and thus interfering with tumor progression, amplifying the roles of autophagy in tumor development and heterogeneity.

## Autophagy as a Mechanism of Protein Secretion

Among the diverse functions triggered by autophagy, “autophagy secretion” has received attention for its ability to alter the secretory profile of the tumor microenvironment, participating in the modulation of processes related to cancer progression (16, 17). Since the term autophagy secretion does not culminate in degradation into lysosomes, we adopt here, like some authors, the term autophagy-dependent secretion (ADS) (18). Nowadays, it is well established that some components of the autophagy route are involved in both conventional and unconventional secretion pathways (19). The conventional secretion route is the best-studied route for protein secretion; it can be regulated positively and negatively by autophagy components, for instance during protein recycling or through the selective clearance of secretory vesicles (20, 21). Unlike proteins exported through the conventional secretory pathway, the cargo delivered to the extracellular space or the plasma membrane by unconventional secretion (UPS) lacks the specific signal peptide and bypasses the classical Endoplasmic Reticulum (ER)-to-Golgi pathways of protein secretion (22). Usually, this pathway is activated by cellular stress, being an alternative route to proteins that use the conventional secretion (23).

Over the last few years, various studies have shown that autophagy takes part in the secretion of several proteins that critically contribute to tumor development. Among them are included different types of cargo, such as High Mobility Group Box 1 (HMGB1), IL1-β and other cytokines, immune mediators, and RNAs (18, 24, 25). Although the major complexes of classical autophagy and their molecular machinery have been clarified, novel and specific autophagy-dependent processes are still under investigation. Despite that, some evidence about the mechanism of intracellular traffic in ADS has emerged by the examination of unconventional secretion in yeast and the alternative route of IL1-β extracellular secretion (**Table 1**) (26, 49). Thereby, the results revealed that some markers involved in this pathway are shared with the classical autophagy program, but there are others exclusive to ADS. Findings of the machinery implicated in cargo selection and release have been suggested three different pathways.

**Table 1:** Evidence summary of studies related to molecular mechanisms and components of autophagy implicated in the three topics covered in this review: secretion, epithelial to mesenchymal transition (EMT) and immunity.

Process	Interfacing mechanisms	References
Autophagy-dependent secretion	TASCC is Rag guanosine triphosphatase–dependent, necessary to recruit mTOR and favor protein secretion. Atg9L and LC3 cooperate to expand the protein secretion compartment. GRASP, Sec, Atg5 and Vsp proteins are required in the process. Specialized SNAREs, as Sec22b and receptors, like TRIM16, coordinate secretory autophagy. ESCRT components also are involved in autophagy secretion pathway.	Narita et al. (20) Duran et al. (26) Dupont et al. (16) Gee et al. (23) Kimura et al. (27) Noh et al. (28) Rabouille (29)
Epithelial - Mesenchymal Transition	TGFβ activates autophagy in early phases of cancer progression; in later phases inhibits ULK1 promoting EMT. Autophagy attenuates EMT by degradation of SNAIL, TWIST and SLUG and activation of ROS-NFκB-HIF1α pathway. ROS-NFκB-HIF1α pathway stimulates SNAIL, N-cadherin expression and thus EMT. Autophagy increase HMGB1 expression and TGF-β/Smad3 signaling enhancing EMT markers. Beclin-1 signaling inhibits EMT by down- regulation of WNT1, ZEB1, and NF-κB. Accelerates EMT increasing Twist and Vimentin.	Gugnoni et al. (30) Zi et al. (31) Wang et al. (32) Li et al. (33) Bao et al. (34) Li et al. (35) Cicchini et al. (36) Catalano et al. (37)
Immunity	LAP: ULK and Rab7 independent. Activated by membrane receptors like TLR2. Dependent of Rubicon and NADPH oxidase (NOX2) to produce ROS and recruit LC3. T cell function involves autophagy activation by TCR or IL-2 receptor, SQSTM1/p62 and Bcl10 degradation. Autophagy controls T homeostasis due expression of ATG3, Vps34, ATG7, ATG5. MHC antigen presentation: Dribbles formation *via* SQSTM1/p62 and LC3. Macrophages differentiation: Involves beclin-1 release from BCL-2 and ATG5 cleavage. Immune cells differentiation and function regulation.	Heckmann et al. (38) Botbol et al. (39) Murera et al. (40) Merkley et al. (41) Munz (42) Zhang et al. (43) Pua et al. (44) Xu et al. (45) Salio et al. (46) Clark and Simon (47) Sil et al. (48)

First, the ATG genes stimulate the generation of an intermediate membrane, not a regular autophagosome, required to LC3I lipidation (LC3II) and the cargo contained within the inner membrane is subsequently delivered extracellularly instead to the lysosomes (50). Second, leaderless proteins are translocated to the intermembrane space of an autophagosome and released directly by fusion with the plasma membrane or with multivesicular bodies (MVBs). The last process proposed consists of an MVB/amphisome intermediate (late endosome-MVB and autophagosome fusion) and the secretion of material in small extracellular vesicles (51).

Considering all strategies, recent studies have been shown that the ADS needs SNARE (soluble N-ethylmaleimide-sensitive fusion protein attachment protein receptors) proteins, as SEC22B, to prevent the fusion with lysosomes and drive the select target to the plasma membrane (52). Besides the regulation by ATG proteins, for instance, ATG5/ATG12/ATG16L1 complex and ATG3, the unconventional secretion also requires of cargo receptors as TRIM (Tripartite motif family) proteins, specifically TRIM16, as well as LC3, GRASP65 (Golgi reassembly-stacking protein)and the GTPase Rab8A of the Ras family, necessary for sorting the target to the plasma membrane (27, 53). Curiously, proteins implicated in extracellular vesicle secretion, like VCP and Rab7, were also found in autophagy pathways and conversely, key ATG proteins, such as ATG12/3 and ATG5, were identified as crucial regulators of exosome biogenesis (54–57). Thus, this data demonstrated a potential link between autophagy and extracellular vesicles (EVs) machinery, processes that contribute to cellular communication and signaling in the tumor microenvironment acting as modulators of tumor progression and aggressiveness. All data collected until now expose the relevance of the set of proteins released by ADS to contribute with some of the hallmarks of cancer (58).

Most of the proteins released by ADS can activate invasion and metastasis, induce resistance to cancer therapies and/or promote inflammation, helping tumor cells to mitigate stress. Such set includes cytokines that use unconventional routes, as TGF-β and IL1-β, both responsible for playing antagonistic roles within the tumor microenvironment, depending on the cellular context (59, 60). Despite that, many studies support their functions as tumor promotors influencing in the inflammatory response of immune cells and contributing to immune suppression, tumor growth, angiogenesis, and metastasis, as observed in breast cancer cells, where the secretion of IL1-β drives colonization of the bone microenvironment, establishing a metastatic niche and cell proliferation (61, 62).

Similar responses were observed in HMGB1 and ATP secretion. Extracellular HMGB1 induces pro-inflammatory cytokines and regulates other genes leading to cell migration and metastasis. HMGB1exerts pro-tumoral functions favoring prostate cancer cells survival and cancer progression (63). Simultaneously, HMGB1 is also responsible for autophagy induction. Regarding ATP, recent studies support its role in tumor survival by switching the ATP-gated receptor P2x to nfP2X_7_, a non-pore functional, that impairs the membrane permeability and the subsequent cell death (64). In addition, IL-6 and IL-8 secreted by autophagy are key determinants of the senescence-associated secretion phenotype (SASP), characteristic of senescence activated by DNA damage-mediated signals (65–67). High levels of both cytokines secreted by tumor cells and other cells such as cancer-associated fibroblasts (CAF) establish a senescent microenvironment and increase tumor aggressiveness showing a correlation with cancer progression and poor prognosis in many tumor types (68, 69). For example, in colorectal cancer, it was showed that peritoneal mesothelial cells control metastasis in SW480 cells and stimulate proliferation by the activation of senescence along with the secretion of mediators as IL-6 and IL-8 (70). Based on these findings and the prominent role of autophagy in cancer, various researchers have hypothesized a link between autophagy and senescence induction (71).

### The Dual Interface of Autophagy and Senescence

One of the first evidence of autophagy acting as an effector mechanism of senescence came from Young et al. (12), who demonstrated, in an oncogene-induced senescence model (OIS), that up-regulation of ATG genes induced autophagy and senescence, while the inhibition of *ATG7* and *ATG5* by shRNA delayed senescence. The OIS program is a dynamic process consisting of an initial phase of rapid proliferation and mTOR activation, a transition phase where diverse changes in morphology, signaling, translation and mTOR activity occur, culminating in a senescence phase, achieved by diverse senescence programs. Thus, autophagy is activated by stress, oncogenic stress, helping to shift the proliferative cell state to a senescent state through the fast protein remodeling and the synthesis/secretion of proteins as IL-6 and IL-8. Later, the same group demonstrated that autophagy is involved in IL-6, IL-8 secretion in a posttranslational manner since the mRNA levels remain stable in ATG knockdown cells. Secretion of these cytokines was further associated with a new type of autophagy called TOR- autophagy spatial coupling compartment (TASCC), which is located at the trans side of Golgi apparatus of senescent cells to accumulate autolysosomes, and mTOR1 facilitating the biosynthesis and secretion of proteins (20, 72). These secretion events were related to survival in tumor cells dependent on autophagy (73, 74). Moreover, several studies in different cell types endorsed the connection between these processes, but the mechanisms are not completely understood and occasionally contradictory, making it crucial to assess what type of autophagy program has been activated (75, 76). Collectively, there is evidence supporting pro-senescence and anti-senescence mechanisms induced by autophagy, including those promoting senescence under different conditions (77, 78).

As a pro-senescence program, a set of studies of Caparelli et al. (79–81), validated an autophagy-senescence transition (AST) process which consists of autophagy activation, metabolism alteration and the subsequent senescence induction in CAFs, responsible to promote tumor growth. They also showed that overexpression of CDK inhibitors (p16/p19/p21) was able to induce autophagy and senescence in CAFs and breast cancer cells favoring tumor promotion. Another study illustrated the notion that p53-mediated senescence is regulated by autophagy, which leads to the degradation of a p53 isoform capable of inhibiting the whole protein, and thereby inducing senescence (82). Likewise, the loss of p53 function can boost SASP in cells and promote tumor growth (83). However, the induction of senescence by wild type p53 has also been reported in different cellular contexts (84, 85). In a different approach, Knizhnik, and collaborators demonstrated that temozolomide triggers autophagy in glioma cells through the generation of DNA adducts, leading to senescence and not apoptosis, thus playing a role in cell survival rather than cell death (86). Besides, exposure of cancer cells to either chemotherapeutic agents or irradiation-induced autophagy is followed by cellular senescence. The entry to senescence has been described as a tumor suppressor mechanism limiting the replication of premalignant cells (75, 87). Although therapy-induced senescence has the intent to suppress cancer cell growth, senescent cells can also contribute with the survival of non-damaged neighboring cells. This protumoral effect of senescence, a bystander effect by SASP activation, may consequently stimulate invasion and tumor progression (88). Alternatively, studies in human fibroblasts showed that autophagy impairment by ATG7, ATG12 knockdown induces premature senescence mediated by activation of p53 and the generation of reactive oxidative species from dysfunctional mitochondria (89). In line with this, a work using a glioma model driven by oncogenic *KRAS* observed that autophagy inhibition using *KRAS:shAtg7* cells predisposes cell to senescence, characterized by β-galactosidase activity and SASP markers (90). A similar outcome came from data of miR-212 in prostate cancer. Interestingly, the authors found that miR-212 is upregulated in benign regions compared with PCa tissues and responsible to negatively modulate autophagy, inducing premature senescence by inhibiting SIRT1. Thus, miR-212 controls senescence induction, acting as a tumor suppressor (91).

Over the last years, senescence has been considered an important process to fight cancer, encouraging the search for anti-cancer therapies based on the induction of cell senescence (92–94). However, studies based on therapy-induced senescence (TIS) indicated the emergence of adverse effects on cancer treatment (95, 96). Chemotherapy-induced SASP drives bone loss in breast cancer and its regulation by p38-MAPK-MK2 inhibition could preserve bone, improving the quality of life of patients (97). TIS may contribute to unwanted outcomes through the stimulation of inflammation by increased secretion of SASP factors, the induction of senescence-associated stemness phenotype or senescence cell scape and further proliferation recovery (98–100). Together, these findings attracted interest to autophagy-modulated senescence and the therapeutic responses associated with both processes since senescence has been implicated with maintaining tumor dormancy, and thus mediating cancer relapse (101, 102). Then, senescence has a potential pro-tumorigenic role supporting aggressiveness, survival responses and shorter recurrence-free survival in patients (103). Finally, regarding its pro-tumorigenic role, there is increasing evidence that SASP components are involved in the establishment of an immunosuppressive environment and in the induction of EMT in TME (104–107). Further studies addressing these novel functions of autophagy and senescence in the tumor microenvironment are warranted and may pave the way to novel targeted therapies that increase the efficacy of the existing cancer treatment modalities.

### The Effects of Autophagy on Epithelial-to-Mesenchymal Transition

Besides being involved in the regulation of protein secretion and tumor cell immunogenicity, autophagy has also been implicated in the process of tumor cell invasion. One of the first associations between autophagy and the invasion process was evidenced by the capacity of epithelial cells to evade anoikis *via* autophagy, what enabled cancer cells migration and invasion (108). More recently, autophagy has been connected to epithelial-to-mesenchymal transition (EMT), a critical multistep process required for cancer cells to invade and metastasize (109, 110). During EMT, epithelial cells undergo profound molecular and biochemical changes to be transiently converted into mesenchymal cells to gain motility, invasiveness, stemness characteristics, and chemoresistance. Multiple embryonic signaling pathways cooperate in the initiation and progression of EMT, including TGFβ, WNT, Hedgehog, and Notch (109, 111).

Notably, there is a multifaceted link between autophagy-correlated and EMT-correlated signaling pathways, reflected by an intricate web of regulatory signaling pathways that converge on the regulation of EMT and autophagy, and that may alter the reciprocal equilibrium between these two processes (30). These pathways often activate EMT-transcription factors and are initially triggered by extracellular signals (112). Probably, the best characterized EMT inducer is TGFβ, known to trigger EMT through the activation of SMAD, PI3K/AKT, MAPK, and Rho-GTPases (112). During cancer progression, cells that undergo EMT require autophagy activation to survive the metastatic spreading. On the other hand, autophagy tends to inhibit the early phases of metastasis, contrasting the activation of the EMT mainly by selectively destabilizing crucial mediators of this process, such as TGFβ (**Table 1**). As part of the tumor-suppressive program dependent of TGFβ, it would promote autophagy in the early phases of tumor formation. On the other hand, later in tumor progression, TGFβ would restrain autophagy while inducing EMT and promoting metastatic spreading of cancer cells (30). Regarding TGFβ and the convergence of signaling pathways between both processes, it was identified recently that the autophagic activity mediated by the transcription factor EB regulates TGFβ signaling in melanoma. Blockage of the BRAFi-induced autophagy function led to an augment of EMT activation and metastasis by enhancing TGFβ signaling, which was responsible for driving tumor progression (113).

Based on the complex relationship between autophagy and EMT, controversies have emerged in the literature regarding the role of autophagy inhibition on EMT: while several studies implicate autophagy in the promotion of EMT, others have suggested the inverse, indicating that inhibition of autophagy could promote EMT and consequently induce cancer cell invasion. Although considerable evidence suggests that the inhibition of autophagy will improve cancer therapy and despite early phase clinical trials show promising results for the use of hydroxychloroquine for this purpose (114), others have highlighted possible undesirable effects of the inhibition of autophagy in cancer therapy (31, 115, 116). Supporting the beneficial effect of autophagy inhibition during cancer progression, there are several compounds and/or microenvironmental conditions that activate the EMT program, and can also induce an autophagic response in different types of cultured cancer and non-cancerous cells, impairing EMT. It has been suggested that EMT impairment could benefit the treatment efficacy of renal cell carcinoma with existing therapeutic regimen when combined to the autophagy inhibitor chloroquine, supporting the evidence that an EMT-like phenotype corresponds to a higher autophagic flux (15). In this regard, it has been also suggested that autophagy is required for EMT induction and metastasis in hepatoblastoma cells (117) and for TGFβ1-induced EMT in non-small-cell lung carcinoma cells (118). Additionally, autophagy induced by starvation was able to activate migration, invasion, and EMT marker expression upon rapamycin induction, and BECN1 knockdown reverted this phenotype. (119). Also, following mTOR signaling inhibition, which is known to induce autophagy, the migration, invasion and EMT marker expression were reduced in colorectal cancer cells (120). Moreover, autophagy is critical for hepatocellular carcinoma cells invasion through the induction of EMT and activation of TGF-β/Smad3-dependent signaling, which plays a key role in regulating autophagy-induced EMT (33), as well as is required for TGFβ2-induced EMT and reactive oxygen species (ROS) modulation in these cells (34). Another model of hepatocellular carcinoma revealed that inhibition of autophagy did not alter cell migration, invasion or EMT marker expression *in vitro*, however sensitized cells to anoikis and decreased lung metastases *in vivo* (121). Therefore, the role of autophagy in EMT seems context-dependent and indicates that the effects of autophagy inhibition in the establishment of metastasis are not necessarily due to its effects on EMT, but rather on its effects on other steps of the metastatic process or in the promotion of cell death. In this regard, autophagic stimulation of metastasis could be simply a consequence of its pro-survival activity against the apoptotic signals coming from changes in adhesion and cytoskeleton reorganization (121).

Ultimate evidence has indicated that autophagy activation could rather induce a reversion of the EMT phenotype and several anticancer compounds that induce autophagy also inhibit EMT (**Table 2**) (37, 122–129, 135). By its dual role in cancer, the effect of autophagy on EMT appears controversial and likely dependent on the cellular type and/or stage of tumor progression (130–134). Thus, at early stages of metastasis, autophagy could inhibit the EMT program mainly by destabilizing EMT crucial players. Later, metastatic cells could require sustained autophagy to survive environmental and metabolic stressful conditions encountered (30). Therefore, our efforts should be concentrated in selecting the precise approaches needed to stimulate or block autophagy in a time/context-dependent manner, to primarily suppress EMT and control cancer progression.

**Table 2:** Examples of autophagy modulation and its role in epithelial-mesenchymal transition (EMT) in cancer.

Autophagy	Cell/tissue	Function	References
**Induction by the overexpression of DEDD (death effector domain-containing protein)**	Breast cancer tissues	EMT inhibition	Lv et al. (122)
**Induction by AZD2014 (mTOR inhibitor)**	Hepatocelullar carcinoma	EMT inhibition	Liao (123)
**Induction by Alisertib (Aurora Kinase A inhibitor)**	Osteosarcoma Colorectar cancer cells	EMT inhibition	Niu (124) Ren (125)
**Induction by Metformin (AMPK activation)**	Thyroid cancer	EMT inhibition	Han et al. (126)
**Induction by Danusertib (Aurora Kinase A/B/C inhibitor)**	Ovarian cancer	EMT inhibition	Zi (31)
**Induction by Rapamycin or PP242 (mTORi)**	Glioblastoma cells	Reverse EMT, inhibit invasion	Catalano et al. (37) Mecca et al. (127)
**Induction by Brusatol (Nrf2 inhibition)**	Hepatocelullar carcinoma	Suppress invasion capacity and EMT	Ye et al. (128)
**Induction by SB202190 and SP600125 (p38-JNKi)**	Ovarian cancer cells	EMT inhibition	Chen et al. (129)
**Inhibition by cloroquine**	Hepatocarcinoma cells	EMT inhibition	Hu et al. (130)
**Inhibition by Cudraxanthone D**	Oral squamous cell carcinoma	Suppress EMT	Yu et al. (131)
**Induction by Rapamycin**	Acidic gastric cancer cells	Antimetastatic effect, Reverse EMT	Wang et al. (132)
**Induction by Cisplatin (DNA damage)**	Nasopharyngeal carcinoma cells	Promote EMT	Su et al. (133)
**Induction by Alteronol (Akt/mTORi)**	Melanoma cells	Promote invasion	Bao et al. (134)

### Autophagy Plays an Essential Role in Metastatic Dormancy

Once the transformation has occurred, autophagy can maintain cellular senescence to avoid the proliferation of transformed cells (109). Accumulating evidence indicates that autophagy is also a fundamental characteristic of stem cells, including cancer stem cells (CSCs). As CSCs are likely to play a central role in tumor dormancy, it appears that autophagy could contribute to the capacity of these cells to survive for extended periods of time in a dormant state and eventually give rise to recurrent tumors that are determinants of morbidity and mortality in cancer patients. Hence, once a tumor is established, tumor cells use autophagy as a survival mechanism to metabolic stress and hypoxia, to maintain tumor-related inflammation, CSCs survival and resistance to therapy.

The ‘reawakening’ of tumor cells at distant sites leading to the outgrowth of metastatic disease many years after primary tumors were treated has led to the concept of metastatic dormancy (136–138). Several studies have shown a role for autophagy in promoting cancer cells survival during dormancy (139). Autophagy may promote the dormancy of disseminated tumor cells simply by supplying key amino acids and other nutrients or, autophagy may play a more instructive role by eliminating mitochondria, modulating redox balance, and actively promoting the CSC state (136, 140, 141). However, it has been suggested that dormant tumor cells are CSCs that depend upon autophagy to survive at distant sites over extended periods of time to expand later as metastatic lesions composed of both CSCs and non-CSCs, representing the full heterogeneity of rapidly growing tumors (136, 139). Indeed, dormant disseminated cancer cells can survive for several years before recurring as extremely aggressive metastatic tumors. There are relevant observations providing insights into the connection between autophagy and dormancy. Despite the autophagy-associated dormancy has not been fully elucidated and some results seem controversial or related to specific phenomena, several studies recognize its crosstalk with cancer relapse (142). Findings in dormant breast cancer cells support that autophagy is crucial to promote their metastasis and survival, probably preventing the accumulation of ROS and damaged organelles (143). Additionally, increased unfolded protein response (UPR) markers have been found in dormant cells. Considering the established link between autophagy and UPR under stress conditions, it has been suggested that UPR-induced autophagy activation in dormant cells to sustain tumor survival (144, 145). Breast cancer stem cells (BCSCs), which undergo tumor initiation and unlimited self-renewal, also exhibit dormancy-associated phenotypes by upregulating autophagy during metastatic dormancy to survive environmental stress and nutrient poor conditions. Consequently, therapeutic targeting of autophagy is actively being pursued as an attractive strategy to alleviate metastatic disease and the recurrence of dormant BCSCs (146).

In conclusion, dormant cancer cells are especially dependent on autophagy for survival, which provides a rationale for combining autophagy inhibition with conventional therapeutic strategies to eliminate these cells and prevent subsequent metastatic outgrowth (147).

### Autophagy and Immune System

As mentioned before, autophagy has an important role in innate and adaptative immunity and can act in several steps of the immune response, leading to its activation or inhibition, depending the context, taking part in tumor immunosurveillance. Besides the ability to modulate the TME through its secretory function, autophagy regulates cellular components (natural killer (NK) cells, dendritic cells (DC), macrophages and lymphocytes (T and B cells)) of immune response, acting on differentiation, proliferation, activation, survival and homeostasis of these cells (**Table 1**). Moreover, autophagy acts on cytokines (interleukins (IL), interferons (IFN), transforming growth factors (TGF)) and antibodies production as well as phagocytosis. Interestingly, cytokines can act as autophagy stimulators or autophagy inhibitors (148).

Autophagy also has a role in tumor response to immunotherapy and a better understand of autophagy-modulation of innate and adaptative immune response could contribute to better strategies to circumvent immunotherapy resistance. For example, autophagy enhances antigen delivery to immune cells (antigen-presenting cells (APCs) and CD8+ cytotoxic T lymphocytes) and in this way can initiate an immune response against tumor cells and enhance immunotherapy efficacy. However, in the case of cancer development, autophagy is a double edge sword for immunity since it can inhibit immune response and attenuate immunotherapy outcomes (149, 150).

### Autophagy and Innate Immunity

Innate immunity is the first defense of eukaryotic cells against invading pathogens and autophagy participates in the process with autophagy adaptor proteins that interact with pattern recognition receptors and activates immune response together with elimination of intracellular invaders (47). The activation of innate immune receptors as Toll-like receptors (TLRs) and nucleotide oligomerization domain-like receptors (NLRs) induces innate-immunity-mediated autophagy upregulation (150–152). TLRs interact with pathogens on the cell surface and usually are also expressed in cancer cells, inducing cytokines production together with NF-κB and MAPK pathways activation (151–153). TLRs are proposed to activate autophagy as demonstrated for TLR2 that enhances innate immunity through ERK and JNK signaling pathways after autophagy stimulation, and also boosts autophagy in glioma cells being correlated with poorer patients outcome (154, 155). For TLR4, the autophagy induction is mediated by TRIF (Toll-IL-1 receptor adapter-inducing IFN)/RIP1 (receptor-interacting protein)/p38-MAPK axis (156). TLR4 and TLR3 activation after LPS (lipopolysaccharides) treatment induces autophagy by TRIF pathway, which contributes to TRAF6 ubiquitination followed by MAPK and NF- κB activation and harmful cytokine production, leading to lung cancer cell migration and invasion (157). In p62 *knockout* cancer cells, stimulation of TLR4 induced activation of the TRAF6-BECN1-autophagy axis leads to cancer cell migration and invasion (158). Additionally, in patients with luminal breast cancer, higher levels of TLR4 and accumulation of LC3II were observed in CAFs. These features were associated with a more aggressive relapse and poorer prognosis in the cohort of patients studied (159). Taken together, TLR and autophagy activation can contribute to tumor development since it enhances survival and proliferation of cancer cells and also triggers the release of cytokines and immunosuppressive factors, contributing to immune evasion and tumor cell resistance (160).

NLR family members, such as NOD1 and NOD2, that recognize intracytoplasmic pathogens, can also activate NF-κB and MAPK pathways and produce immunosuppressive cytokines, as well as induce autophagy by recruiting ATG16L1 (161, 162). Both NOD1 and NOD2, altering the balance of anti- and pro-inflammatory cytokines, can modulate the risk of cancer development (163). For example, in triple negative breast cancer (TNBC), the expression of NOD1 and NOD2 is associated with cancer progression and a global proteome profiling of TNBC-derived cells overexpressing these receptors demonstrated disrupted immune-related pathways such as NF- κB and MAPK signaling and autophagy (164).

### Autophagy and Adaptive Immunity

Autophagy participates in adaptative immune response such as thymus selection, lymphocyte development and homeostasis, antigen presentation and cytokine release, exerting anti-tumor effects (47, 165). Adaptative immunity occurs when extracellular or intracellular peptide epitopes are presented by APCs through the major histocompatibility complex (MHC) class I and II to CD8+ and CD4+ T cells, respectively. The interaction of antigen and T cell receptors triggers cellular (cytotoxic lymphocytes) and humoral (antibody-producing B cells) adaptative immune response (42, 166). The efficient antigen presentation requires proteasomal or lysosomal antigen degradation and delivery of resulting peptides to MHC molecules and this step can be enhanced by autophagy, for example, in APCs upon uptake of extracellular antigens (e.g. tumor antigens) and in antigen processing for MHC I cross- presentation. Autophagosomes facilitate intracellular trafficking of these antigens to endosomes to be degraded by cathepsins followed by peptide load onto MHC II molecules that mature and get translocated to the plasma membrane and present antigens to CD4+ T cells (42, 166). A non-canonical regulation of phagocytosis by ATG proteins can also be used to engulfment of extracellular antigens, which is known as LC3-associated phagocytosis (LAP), characterized by a single membrane vesicle decorated with LC3-II instead of double-membrane autophagosome as in autophagy (**Table 1**) (42, 167).

### Autophagy and Immune Cells

Autophagy has multiple roles on immune cells acting during their differentiation, proliferation, activation, and homeostasis maintenance and in this setting can also promote or inhibit tumor development (150). Dendritic cells link the innate and adaptative immune system as they are powerful professional APCs. Autophagy is involved in different DC functions both in physiological and pathological conditions (168). The inhibition of autophagy impacts the ability of DCs to process and present cytoplasmic antigens through the MHC II pathway and cytokines secretion, which increases their immunostimulatory phenotype (169–171). Macrophages are also APCs that require autophagy during the differentiation process in monocytes from the bone marrow into macrophages in tissue site (43, 172). Granulocyte-macrophage colony-stimulating factor (GM-CSF) is a signal to maturation and prevents monocytes apoptosis together with autophagy induction. When autophagy is downregulated either by BECN1 knockdown or pharmacological inhibition using 3-methyladenine (3-MA) and chloroquine, caspases are activated and cytokine production is prevented (43). Autophagy is also involved in macrophage polarization with its inhibition leading to the classical activation profile and augmented pro-inflammatory cytokines secretion, and its induction promoting macrophage alternative activation, resulting in increased production of anti-inflammatory cytokines (173).

T cells use basal autophagy to maintain organelle homeostasis and it can be induced after T cell antigen receptor (TCR) stimulation. Moreover impaired autophagy after deletion of ATG proteins (ATG3, ATG5, and ATG7), BECN1 or Vps34 can hinder T cell survival, proliferation, differentiation, and activation (150, 174–176). Autophagy proteins may also be involved in other functions besides autophagy as demonstrated for memory CD8+ T cell, in which UVRAG (ultraviolet radiation resistance-associated protein) deletion does not impair autophagy but affects proliferation (177). On CD4+ T cells, autophagy impairment after BECN1 deletion leads to apoptosis upon TCR stimulation (178). On the other hand, blockage of mTOR signaling after rapamycin treatment in effector CD8+ T cells can enhance memory CD8+ T cells in lymphoid tissue or inhibit them in mucosal tissue (179). In antigen-specific memory CD8+ T cells, deficient autophagy leads to the accumulation of damaged mitochondria and increased apoptosis (180). Moreover, mTOR status can also interfere with T cell differentiation since its induction lead to activated T cell to differentiate into Th cells and its downregulation together with AMPK induction cause naïve T cells differentiation into regulatory T (Treg) cells (181). The metabolic profile also influences the dependence on autophagy since cells as memory lymphocytes and Treg cells, that use more oxidative phosphorylation (OXPHOS), are more dependent on autophagy for homeostasis than effector T cells that use preferentially aerobic glycolysis (45, 181, 182). On Treg cells, impaired autophagy induces mTORC1 and MYC signaling pathways, contributing to apoptosis induction (182).

For B cells, autophagy participates during cell development to support extremely high metabolic demands for their differentiation and reaches maximal levels during the earliest stages of development and diminish as B cells mature. It is also important, at basal levels, to maintain peripheral B cell numbers as required to cell survival after LPS stimulation, as well as for IgM production after immunization. However, autophagy is not essential for transition of pro- to pre-B cell stages in the bone marrow and B cell activation after BCR stimulation (183). Mature B cells with impaired autophagy (Atg5-/-) accumulate damaged organelles and have enlarged endoplasmic reticulum together with ER stress, more antibody secretion and plasma cells apoptosis (184).

Autophagy is also required for NK cell differentiation, since it regulates the number and quality of mitochondria on proliferating NK cells and enhances memory NK cells in an ATG3 dependent manner (185). In invariant natural killer cells (iNKT), it is observed a high level of autophagy during iNKT cell thymic differentiation into memory cells to regulate mitochondrial content and ROS production. A conditional deletion of Atg7 gene in T-cell compartment blocked iNKT development and maturation, as well augmented its susceptibility to apoptosis (46). In another study, the deletion of Atg5 or Atg7 decreased iNKT mature cells and IL-4 and IFN-γ levels accompanied by an increase in apoptosis (186).

In neutrophils, autophagy deficiency has no impact on their morphology, migration, granular content, apoptosis or effector functions, but in autophagy-deficient mice, neutrophil proliferation and differentiation is augmented, indicating an inverse correlation between autophagy and neutrophil differentiation (187).

As mentioned previously, there are feedback loops between autophagy and different cytokines. For IL-1 (IL-1α and IL-1β), autophagy limits its secretion although it is observed autophagy induction by these cytokines, indicating a negative feedback mechanism (188, 189). The interferon family (IFN types I and II) also induces autophagy in epithelial, immune, and tumor cells (190, 191). IL-2, IL-12, and TGF-β also stimulate autophagy (192–194). On the other hand, IL-6 has an anti-autophagic effect in starvation-induced autophagy in U937 cells (195) but stimulates autophagy in B cells (196). IL-10 also inhibits starvation-induced autophagy in DCs (197).

Autophagy can also act in immune tolerance mediated by immunotherapy strategies since immunologic molecules such as indoleamine 2,3-dioxygenase (IDO), PD-1 and CTLA-4 can be regulated by autophagy pathways. IDO is found in tumor sites and has anti-tumor immunity effects through interference with a cytotoxic T-cell response, DC maturation and increase in Treg population, promoting immunologic tolerance and tumor development, but its production can be inhibited by autophagy stimulation (198, 199). PD-1 from tumor cell surface interacts with PD-L1 on T-cells and acts as an inhibitory checkpoint molecule, preventing recognition of tumor cells, suppressing T cell proliferation, development, and anti-tumor immunity. It has been reported that the interaction of PD1 with its ligand limits nutrients availability to nearby T-cells, promoting autophagy induction (200). Treatment with Sigma1 can induce autophagy in co-cultured T-cells and tumor cells, leading to degradation of PD-L1 and suppression of PD-1 and PD-L1 interaction, which could favor immunotherapy effects due to immune microenvironment modulation (201). However, the expression of PD-L1 affects several genes involved in mTOR signaling and autophagy. When PD-L1 is lost, it is observed autophagy upregulation and less sensitization to autophagy inhibitors to reduce tumor cell proliferation (202). CTLA-4 is another immune tolerance checkpoint and an effective target for tumor treatment. In human melanomas, over-expression of MAGE-A, a cancer-germline antigen, is associated with CTLA-4 blockade resistance and can downregulate autophagy, suggesting autophagy induction as a potential therapeutic approach to improve CTLA-4 inhibitors efficacy (203).

### Autophagy in Immune Cells: Dual Functions Shaping Tumor Response

Increasing data suggest that autophagy can interfere with antitumor immunity together with tumor development and survival (149, 150). Knockdown of ATG5 in cancer cells was followed by increased induction of DC maturation, production of IL-6 and IFN-γ along with the proliferation of CD4+ and CD8+ T-cells after an immunogenic cell death inducer treatment (204). In Treg cells, autophagy is an important and active process to support their homeostasis contributing to their immunosuppressive profile. Suboptimal NK cell activity induces autophagy in surviving tumor cells, leading to treatment resistance (205). As mentioned before, many pro-inflammatory cytokines contribute to tumor growth, metastasis and can induce autophagy (206). Although the treatment with high-dose-IL-2 has antitumor effects it is limited by severe side effects, as multiorgan dysfunction that is accompanied by systemic autophagic syndrome induced by cytokines. In a murine model of metastatic liver tumor, the combined treatment of high-dose-IL2 and chloroquine increased antitumor effects along with decreased toxicity, increased long-term survival and enhanced infiltration of immune cells in liver (207). In a renal cell carcinoma model, autophagy inhibition also improved HDIL-2 anti-tumor effects due to apoptosis induction and immune system stimulation together with increased activity of DCs, T-cells, and NK cells (208). Inhibition of autophagy through 3-MA treatment also potentiates apoptosis induced by IL-24 in oral squamous cell carcinomas, demonstrating that autophagy inhibition can be explored as a promising approach to increase immunotherapy efficacy (209). The phytochemical shikonin can induce necroptosis accompanied by autophagy enhancement that directly contributes to DAMP upregulation in tumor cells. However, if autophagy flux is blocked by chloroquine treatment, there is an even greater upregulation of ectoDAMPS, resulting in DC activation. In the context of DC vaccines, the pretreatment of tumor cells with chloroquine and shikonin potentiated antimetastatic activity and reduced chemotherapy doses *in vivo* (210). Autophagy can also reduce immunotherapy effect by impairing cytotoxic T-lymphocyte (CTL)-mediated tumor cell lysis when autophagy is induced under hypoxia conditions, activating STAT3 signaling in target cells which in turn favors tumor cell survival, proliferation, and immune escape. If autophagy is blocked in this context and CTL-response is boosted with a vaccination strategy, vaccination efficacy is improved, leading to tumor regression *in vivo* (211). Hypoxia-induced autophagy also impairs NK cells function by degradation of NK-derived granzyme B in autophagosomes of hypoxic breast cancer cells, leading them less susceptible to NK killing and immunotherapy effects. However, if autophagy is blocked by deletion of BECN1, granzyme B levels are restored and favors tumor regression *in vivo* due to tumor cell death by NK-mediated lysis (212). Pancreatic ductal adenocarcinoma (PDAC) is known for immune checkpoint blockade resistance and frequently altered MHC-I expression that facilitates immune evasion trough NBR1 selective autophagy downregulation of MHC-I. If autophagy is inhibited, there is recover in MHC-I expression and augmented immunotherapy response along with enhanced T-cell immunity in tumor models *in vivo* (213). In a cohort of gastric cancer (GC) patients, the expression of CXCL10 has a positive correlation with patient prognosis and induces T lymphocyte migration and infiltration into the GC 3D cell culture model. It is also observed in basal conditions that GC cells have increased autophagy, and the knockdown of essential autophagy genes (Atg5 and Atg7) or their pharmacological block in these cells augmented CXCL10 expression under normal and hypoxic conditions facilitating T cell lymphocyte migration and potentiating tumor immunity (214). Recent studies demonstrated that autophagy activation in tumor cells is one of the main reasons for decreased antitumor immune response, reinforcing the concept of autophagy inhibition as a valuable approach to increase immunotherapy results. One of the autophagic proteins that have been recently described as drug targetable is Vps34, whose inhibition with genetic or pharmacological approaches decreased tumor growth along with increased mice survival due to infiltration of immune cells (NK, CD8+ and CD4+ T effector cells) within the tumor microenvironment, which could turn cold tumors into hot inflamed tumors to enable immunotherapy treatments. Moreover, the combined treatment of Vps34 inhibitor and anti-PD-L1/PD-1 in melanoma and colorectal cancer models prolonged mice survival and enhanced immunotherapy benefits (215). Impairment of autophagy with BECN1 ablation is also beneficial to increase NK infiltration and inhibit tumor growth in a melanoma tumor model. In addition, NK infiltration in the tumor microenvironment is mediated by CCL5 chemokine overexpression in autophagy-deficient cells trough c-Jun/JNK activation. Similar results were also obtained after deletion of other autophagic genes as *Atg5* and SQSTM1/p62 and pharmacological inhibition by chloroquine. In conclusion, targeting autophagy may be a valuable approach to improve immunotherapy mediated by NK cells (216).

On the other hand, autophagy has also anti-tumoral effects since its induction contributes to a better response of helper T lymphocytes (HTLs) against head and neck squamous carcinoma cells and its inhibition decreases HTLs recognition of tumor cells (217). Tumors can act as antigen donor cells that require autophagy to form tumor-derived autophagosomes (Dribbles) which contain tumor-associated antigens. Dribbles can stimulate efficient cross-presentation of T-cells (218) and induce B cell activation along with cytokine release and antibody production (219) which can contribute to tumor control and elimination. For vaccination strategies, in contrast to whole-cell tumor vaccine, Dribbles prime T cells by enhancing costimulatory molecules as well as MHCI, and reduce tumor formation on hosts challenged with nonhomologous tumors, effect limited if there is depletion of the autophagic protein SQSTM1/p62 (220, 221). For efficient immunotherapy, tumor antigens should be immunogenic, essential only for tumor cells and overexpressed in tumors compared to normal tissue. Recently, it was demonstrated that SQSTM1/p62 fits all these requisites and a DNA-vaccine encoding this protein had antitumor and antimetastatic effect against several tumor models in dogs, suggesting that this can be a useful strategy for immunotherapy (222). Inactivation of ATG5 in non-small cell lung carcinoma favors carcinogenesis and its development is accelerated in KRASAtg5(fl/fl) autophagy-deficient mice. In these mice, a higher expression of ENTPD1/CD39 was observed, culminating in an immunosuppressive environment along with increased Treg infiltration that contributes to tumor development (223). As discussed previously, autophagy plays a role in monocytes/macrophages recruitment, what decreases infiltration in liver tissues accompanied by autophagy reduction and hepatocarcinogenesis (224). Immunotherapy can also be potentiated by autophagy. For instance, in murine tumor models, the treatment with chemotherapy or radiotherapy induced autophagy, which favored translocation of the mannose-6-phosphate receptor (MPR) from autophagosomes to tumor cells surface, rendering the cells more sensitive to granzyme B from activated CTLs, potentiating CTLs killing and immunotherapy (225, 226). In another example, autophagy elicited by alpha-tocopheryloxyacetic acid (α-TEA) on tumor cells improved cross-presentation of tumor antigens for MHCI and MHCII, which can be use as an adjuvant strategy to improve anti-tumor immune responses and strength immunotherapy (227, 228). Treatment of ovarian cancer models with farletuzumab, a humanized monoclonal antibody against folate receptor α, also induced autophagy and reduced proliferation, which was reversed by autophagy pharmacological blockage (229). Finally, studies indicate that although statins have no protective effect on breast cancer incidence, they can be used as adjuvant therapy to increase apoptosis and radio-sensitivity along with proliferation and invasion inhibition of cancer cells. Fluvastatin belongs to the statin family and when used *in vitro* to treat breast cancer cells, it induced autophagy but with impaired lysosome function which may contribute to cell death. Moreover, a decrease of pro-inflammatory cytokines, such as IL-6 and TNF-α, was observed along with autophagy consequent effect in tumor immunity (230).

## Concluding Remarks

**Figure 2:**
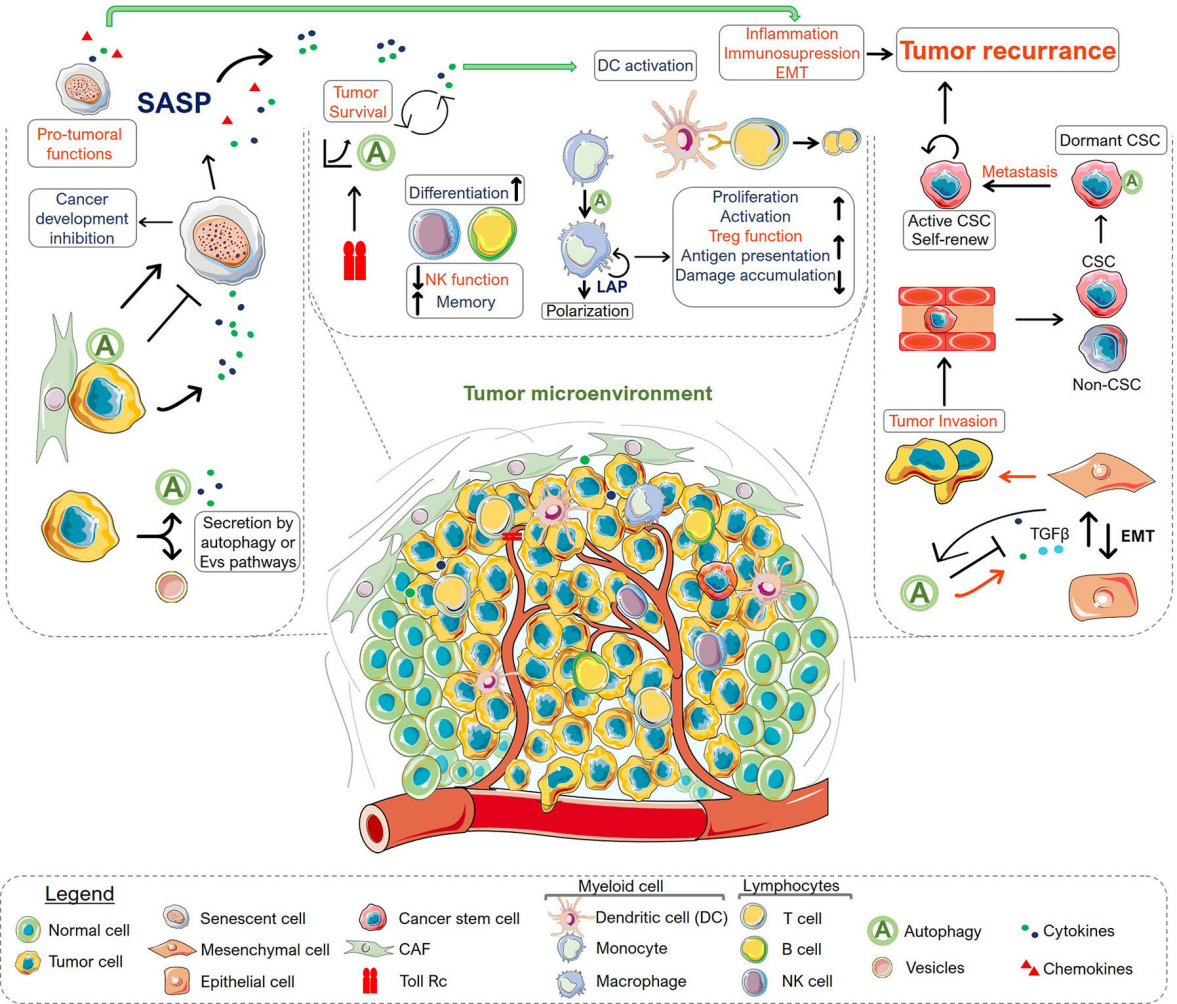
Overview of autophagy roles in the tumor microenvironment. The scheme summarizes the role of autophagy in secretion (left), immune system (middle), epithelial-mesenchymal transition (EMT) and tumor dormancy (right). There is an intricate and dynamic network of signaling circuits that drive tumor development and progression within the tumor microenvironment. The connectivity among various processes may regulate the fate of the microenvironment components, indicating the importance of viewing this as an emerging system, where the resulting interactions are larger than the sum of the individual parcels. Autophagy can act in many ways in different types of cells displaying anti-tumoral (shown in blue) or pro-tumoral functions (shown in red). Protein secretion by CAF or tumor cells can modulate cellular states inducing or inhibiting senescence, which ultimately can control tumor survival, immune cell response and interfere with the epithelial-mesenchymal transition, affecting tumor invasion capacity. In the context of the immune system, autophagy has a key role in immune cell differentiation, proliferation, activation and effector function, covering the range of homeostatic to reactive functions of the immune system. At the same time, autophagy is also connected with the innate immune response being controlled by receptors such as TLRs. Importantly, in advanced stages, the autophagy system in tumor cells is involved with EMT and the consequent ability of cancer cells to invade tissues and metastasize. The interplay among these functions contributes to tumor aggressiveness. Moreover, autophagy was also appointed as a characteristic of cancer stem cells (CSC) playing a central role in tumor dormancy. Altogether, the myriad of connected process regulated by autophagy in the TME modulate tumor response and may determine its regression or progression. Altogether, understanding the integrated mechanisms that regulate autophagy within the TME constitute a niche for development of novel strategies for combination therapy.

Given the dual role of autophagy in cancer and its involvement in cancer therapeutic responses, the process of autophagy has been pointed as an important theme in cancer research (**Figure 1**). The interconnection of autophagy to the regulation of several biological processes in the TME indicates that autophagy has key roles in tumor progression. Thus, in addition to its primary function of degradation and recycling, most of the components of the autophagy machinery also mediate numerous non-autophagic functions. This suggests that autophagy operates in many ways, establishing an intricate network of signaling, along with other cell elements, integrating diverse signals within the TME and regulating the fate of cancer and other microenvironmental cells (**Figure 2**). Integrative approaches, which address the impact of autophagy inhibition in complex systems, are therefore necessary for the development of strategies that exploit the autophagy machinery as a target to control tumor growth, without impeding the generation of a long-lasting memory cytotoxic immune response or the induction of a stemness phenotype in residual cancer cells. Models of the intricated and dynamic network of cancer cells and the tumor microenvironmental cells are warranted for filtering compounds that may control tumor growth and increase the efficacy of many known therapeutic regimens.

## Author Contributions

SB, FA, MR, and RC wrote the manuscript. SB drew the figures. SB and RC designed and edited the review. All authors contributed to the article and approved the submitted version.

## Funding

The authors’ work has been funded by grants from Fundação de Amparo à Pesquisa do Estado de São Paulo (FAPESP 2014/03742-0 and 2015/22814-5) and Conselho de Desenvolvimento Científico e Tecnológico (CNPq 305700/2017-0).

## Conflict of Interest

The authors declare that the research was conducted in the absence of any commercial or financial relationships that could be construed as a potential conflict of interest.
